# Energy Metabolism during Anaerobic Methane Oxidation in ANME Archaea

**DOI:** 10.1264/jsme2.ME16166

**Published:** 2017-03-17

**Authors:** Shawn E. McGlynn

**Affiliations:** 1 Earth-Life Science Institute, Tokyo Institute of TechnologyOokayama, Meguro-ku, TokyoJapan; 2 Biofunctional Catalyst Research Team, RIKEN Center for Sustainable Resource ScienceWako-shiJapan; 3 Blue Marble Space Institute of ScienceSeattle, WAUSA

**Keywords:** ANME archaea, methane oxidation, methanogenesis, acetate oxidation, extracellular electron transfer

## Abstract

Anaerobic methane oxidation in archaea is often presented to operate via a pathway of “reverse methanogenesis”. However, if the cumulative reactions of a methanogen are run in reverse there is no apparent way to conserve energy. Recent findings suggest that chemiosmotic coupling enzymes known from their use in methylotrophic and acetoclastic methanogens—in addition to unique terminal reductases—biochemically facilitate energy conservation during complete CH_4_ oxidation to CO_2_. The apparent enzyme modularity of these organisms highlights how microbes can arrange their energy metabolisms to accommodate diverse chemical potentials in various ecological niches, even in the extreme case of utilizing “reverse” thermodynamic potentials.

In the 1970s, methane consumption in marine anaerobic environments was observed to be linked to sulfate reduction (6, 55, 76), and this ushered what has become a history of inquiry into the biological phenomena responsible for anaerobic methane oxidation. Early on, an ability of sulfate-reducing bacteria (SRB) to co-metabolize methane was proposed (76), though later it was hypothesized that methanogens might be able to operate in the direction of methane oxidation when paired with H_2_-consuming SRB (114). Field evidence for this was provided with the observation that sulfate stimulated methane oxidation and 2-bromoethanesulfonic acid (an inhibitor of methanogens) inhibited the process, and it was proposed that anaerobic methane oxidation by archaea phylogenetically related to methanogens might occur through a pathway of “reverse methanogenesis” (39).

The subsequent findings of consortia comprised of phylogenetic relatives of methanogens and Deltaproteobacteria (12) which were isotopically depleted in ^13^C (70, 71), appeared to strongly support this hypothesis, and later studies identified a near complete methanogenesis pathway within members of the anaerobic methane-oxidizing archaea ANME-1 clade (34), adding further support to the idea that these archaea were operating the methanogenesis pathway in reverse. Thus it seemed that a syntrophy of methane oxidation coupled to sulfate reduction based on the exchange of reducing equivalents had been identified. A major stumbling block with the idea of methanogens operating in reverse has existed though, since it remained unclear how energy could be conserved during the reversal of an energy conserving process. Said another way, because methanogens make a living by producing methane, just how could it be that methane oxidizing archaea could make a living by consuming it?

## Methanogen or methanotroph? Follow the electrons

Anaerobic methane-oxidizing archaea have been found to be polyphyletic in trees drawn from rRNA and protein encoding functional genes such as Mcr (for an example, see [42]). Therefore, in this paper “ANME” will be used simply to refer to the physiology of methane oxidation in ANaerobic MEthane oxidizing archaea. Where a specific phylogenetic group can be specified, it will be with the associated number and letter combinations used to refer to these clusters (e.g. ANME-1, ANME-2, AOM Associated Archaea (AAA), and AAA cluster member “*Ca.* Methanoperedens nitroreducens).

**Fig. 1:**
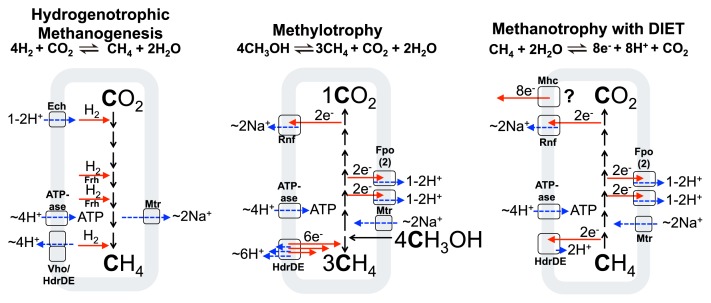
Varieties of physiology in methane metabolising archaea, with respective chemiosmotic coupling steps indicated. Red arrows represent electron transfer, while dashed blue arrows show chemiosmotic ion movement. Enzyme abbreviations and references for the enzymes indicated are: Frh, F_420_-reducing hydrogenase (2); Ech, energy-conserving hydrogenase (101, 112); Vho, methanophenazine-reducing hydrogenase (22); Fpo/Fqo, F_420_H_2_: phenazine/quinone oxidoreductase (7); HdrDE, heterodisulfide reductase (21); Mhc, Multiheme cytochrome (57); Rnf, Na^+^-translocating, ferredoxin:NAD oxidoreductase (88); Mtr, Na^+^-translocating methyl-H_4_MPT:coenzyme M methyltransferase (9, 45).

ANME archaea have evaded attempts of isolation in pure culture, and their slow doubling times (weeks to months [20, 32, 43, 57, 59, 66, 72]) have hampered their investigation. Current thinking on their physiology is guided by knowledge of methanogens, which have been systematically studied in detail for decades (for reviews, see [16, 98, 100, 111]) (Fig. 1). Given that methane-oxidizing archaea appear to exhibit the same central carbon metabolism as methanogens (34, 35, 61, 107), key questions to address in terms of their physiology are i) how they generate ATP from the reverse of methanogenesis, ii) and where the electrons from methane go.

### Methanogen metabolism in comparison to archaeal methanotrophs

In order to understand archaeal methane oxidation, it is useful to discuss the physiology of methane formation in methanogens. Methanogenesis from CO_2_ and H_2_ in hydrogenotrophic methanogens with cytochromes (those that transfer electrons into and out of a membrane redox pool) occurs as shown schematically in Fig. 1 (left) for *Methanosarcina barkeri* (adapted from [100]). There we can see the operation of the following energy converting protein machines: Ech hydrogenase, which is a redox active chemiosmotic pump capable of interconverting chemiosmotic and electronic potentials (101, 112), the methyl-transferring Mtr protein which interconverts the chemical potential of methyl group transfer to the pumping of sodium ions to the outside of the cell (9, 45), the Vho hydrogenase (22)/Hdr heterodisulfide reductase system (21) which couples the movement of electrons and protons to the generation of chemiosmotic potential by a redox loop mechanism (21, 23, 93), and ATPase, which converts the membrane proton/sodium (87) potential back into chemical potential in the form of a high cellular ATP:ADP ratio.

In methanogens which consume carbon compounds of intermediate redox states, such as methanol or methylamines, the carbon pathway is a disproportionation, and the cell forms 3 CH_4_ and 1 CO_2_ for every 4 CH_3_OH consumed to achieve redox balance. The 6 electrons derived by oxidation of 1 CH_3_OH to CO_2_ are used during (three) 2 electron reductions of 3 CH_3_OH to CH_4_. An example of this is shown in Fig. 1 (center) for *M. acetivorans*. Hydrogenase enzymes are absent (although methylotrophic cells such as *M. mazei* that use H_2_ as an electron intermediate exist), and the redox active Rnf (88) and Fpo (7) proteins pump Na^+^ and H^+^ ions out of the cell respectively, while reducing the membrane-bound electron carrier methanophenazine (MP). The reduced methanophenazine from these steps is later reoxidized by the activity of HdrDE, which supplies electrons for methane generation and releases protons from methanophenazine to the outside of the membrane, thereby closing the redox balance and contributing to the proton motive force.

### ANME Archaea: Where do the electrons go?

Methane oxidation with H_2_ as an electron vent (protons as the electron acceptor) has not been supported by genomic (4, 35, 61, 107) nor microcosm studies (60, 63, 66, 67). An alternative proposal was that methane-oxidizing archaea themselves function as sulfate reducers during methane oxidation and produce HS_2_^−^, which could then be used by partner sulfur disproportionators (62). The physiology in that case may be interpreted to operate non-syntrophically, since the amount of energy from sulfate reduction would obviate the need for a syntrophic partner (62). It is not clear how widespread archaeal sulfate reduction with methane is however, since subsequent investigations (108, 109) reported that dominant SRB partner organisms did not grow on elemental sulfur as proposed and microcosm-derived SRB did not have the ability to disproportionate sulfur at the 7:1 stoichiometry of sulfide and sulfate, as previously reported (62).

Other than syntrophic coupling through molecules, there is the possibility of syntrophic coupling through direct electron exchange. Indeed, numerous laboratory studies have reported direct interspecies electron exchange (DIET) between bacteria and also between bacteria and archaea (33, 50–52, 65, 77, 80, 81, 96). Analysis of the ANME-1 draft genome indicated the presence and expression of a number of multiheme cytochrome proteins (61), which led the authors to suggest the possibility of DIET. Support for this was provided by independent investigations of SRB paired ANME-1 and ANME-2 consortia. For ANME-1, electronic coupling was suggested to be facilitated by nano-wire-like structures (see [52, 92] for reviews) that may emanate from the bacterial partner (108), whereas conductivity was suggested to occur for ANME-2 members through a conductive mesh of multi-heme cytochrome proteins that extend from the archaeal S-layer and appear to allow electronic contact with partner SRB (57). These proposed mechanisms await direct experimental confirmation, for example, by conductivity measurements of whole consortia.

One way to test the hypotheses of syntrophic coupling by DIET would be to determine if the syntrophy could be decoupled by adding an appropriately poised electron donor or acceptor which would electronically interrupt the partnership. In the case of ANME-1-targeted experiments, SRB partners were shown to decouple growth from their archaeal syntrophic partners when H_2_ was supplied (108). However, H_2_ appears to only decouple in the case of thermophilic consortia and not in their mesophilic relatives, and this may explain why previous studies failed to observe effects with H_2_ (60, 66, 67, 109). In the case of de-coupling through an added electron acceptor, a number of soluble electron acceptors have been used to demonstrate that methane oxidation by marine ANME-2 can be de-coupled from bacterial partners (84). Addition of oxidized 9,10-anthraquinone-2,6-disulfonate (AQDS) or Fe^III^-citrate resulted in bacterial partners which were dramatically less anabolically active, whereas the partnering methane-oxidizing archaea exhibited similar activity and methane oxidation was sustained. The ability to donate electrons to Fe^III^ and AQDS is characteristic of well-studied microbes involved in DIET (13, 18), and is consistent with previous findings of metal oxides promoting methane oxidation (8). A recent study (27) indicated that iron reduction coupled to methane oxidation was broadly distributed, being accomplished also by “*Ca.* M. nitroreducens”-like members of the phylogenetically distinct AOM-associated archaea (AAA) (42), which also contain protein encoding DNA sequences for large multiheme cytochromes (41, 57).

Other than methane-oxidizing archaea which appear to use Fe^III^ or SRB as a terminal electron acceptor (through sulfate reduction), methane-oxidizing archaea have also been shown to utilize nitrate. This possibility was first revealed when a freshwater consortium of archaea and bacteria was identified that depended on nitrate as an electron acceptor instead of sulfate (74). Astoundingly, this was later found to contain two organisms capable of anaerobic methane oxidation: one bacterial (which is not discussed here and uses nitrite as the terminal electron acceptor [25, 26]), and one archaeal which uses nitrate. These denitrifying anaerobic methane-oxidizing (DAMO) archaea include those now known as “*Ca.* M. nitroreducens” (35), and are also sometimes referred to as ANME-2d (although “2d” may result in confusion with the phylogenetically distinct marine ANME-2a/b/c groups and is avoided here in favor of “DAMO”, “AAA-cluster ANME”, or specifically “*Ca.* M. nitroreducens”). These findings have recently been advanced by a metagenomic analysis of “*Ca.* M. nitroreducens” relatives, which indicated that at least some relatives might use both nitrate and nitrite as an electron acceptor (4).

One critical area moving forward will be to determine if the methane oxidizing archaea apparently capable of electron export to the outside of the cell are actually electronically conductive, since the previous reports inferred this indirectly (57, 108). Research also remains to be done on the multiheme cytochrome proteins proposed to relay electrons to partner SRB or iron oxides, since cytochromes alone have not been demonstrated to be sufficient to support DIET (48, 92). Are the proposed proteins actually expressed? Where are they physically located? What are their electronic mid point potentials? These are all urgent questions to assess the hypothesis of DIET put forward.

A final mystery to be addressed with the above hypotheses are the ANME who apparently live without a syntrophic partner and do not respire nitrate. The metabolism of methane oxidation with electron export might account for those ANME who live in direct contact with a syntrophic partner or have a continuous supply of an oxidant (*e.g.* Fe^III^); however the repeated observation of aggregates comprised only of ANME cells (70, 71, 79) might be a sign of surprises yet to be uncovered.

## Beyond “reverse methanogenesis”: proposed models of energy conservation in methane-oxidizing archaea

The above discussed findings indicate at least three modes of archaeal anaerobic methane oxidation based on the reduction of i) SRB ii) metal oxides such as Fe^III^-citrate, and/or iii) nitrate. But how do the electrons get from methane to these acceptors, and how is energy conserved along the way?

### Proposed metabolism of methane oxidation coupled to extracellular electron acceptors including SRB

**Fig. 2:**
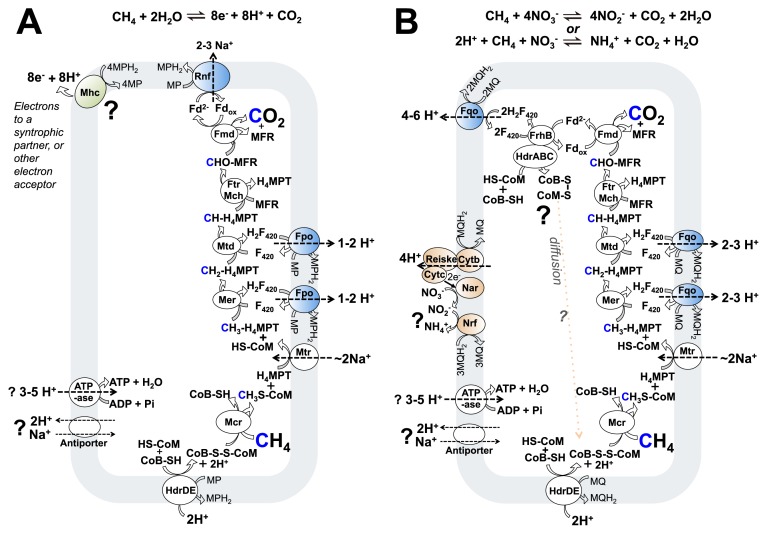
Suggested pathways for the central carbon and energy metabolism of anaerobic methane-oxidizing archaea. A) Archaeal methane oxidation coupled to extracellular electron acceptors and SRB, re-drawn from (57). B) Nitrate-utilizing “*Ca.* Methanoperedens nitroreducens” re-drawn from (4). The cell membrane is indicated in light gray. Enzymes in white are common between archaeal methanotrophs and methanogens, enzymes in blue are found in both archaeal methanotrophs and methylotrophic methanogens, enzymes in green are so far only in archaeal methanotrophs implicated in DIET and Fe^III^ reduction, and enzymes in orange are in nitrate-reducing archaeal methanotrophs only. Protons and sodium ions with chemiosmotic relevance are shown. Mch and Ftr are shown acting in one step only to save figure space. B) shows the possibility of reducing nitrate completely to ammonium, and also two possibilities for oxidizing the HS-CoM and HS-CoB co-factors (discussed in the text). Fqo with respiration on nitrate was proposed to result in more ion pumping than Fpo in methanogens, as discussed in the text. Abbreviations for enzymes and co-factors in the figures are: F_420_, coenzyme F_420_; H_4_MPT, tetrahydromethanopterin; HS-CoB, coenzyme B; HS-CoM, coenzyme M; MFR, methanofuran; MP, methanophenazine; Fd, ferredoxin; Frh, F_420_-reducing hydrogenase; Ech, energy-conserving hydrogenase; Vho, methanophenazine-reducing hydrogenase; Fpo, F_420_H_2_: methanophenazine oxidoreductase; Fqo, F_420_H_2_: quinone oxidoreductase; Hdr, heterodisulfide reductase; Cyt, cytochrome; Nar, nitrate reductase complex; Nrf, nitrite reductase; FrhB, F_420_ hydrogenase subunit B; Rnf, Na^+^-translocating, ferredoxin:NAD oxidoreductase; Fmd, formyl-methanofuran dehydrogenase; Ftr, Formylmethanofuran/H_4_MPT formyltransferase; Mch, methenyl-H_4_MPT cyclohydrolase; Mtd, F_420_-dependent methylene H_4_MPT dehydrogenase; Mer, F_420_-dependent methylene-H_4_MPT reductase; Mtr, Na^+^-translocating methyl-H_4_MPT:coenzyme M methyltransferase; Mcr, methyl-coenzyme M reductase.

For those methane oxidizing archaea which reduce SRB or metal oxides, the suggested (57) model of energy metabolism drew on extant genomic knowledge of ANME-2a (107) and a genomic bin of ANME-2b (Fig. 2A). This model or a close variant may also correspond to the physiology employed in ANME-1 and also during iron oxide reduction by some “*Ca.* M. nitroreducens”. The half reaction for the metabolisms would be:

(1) $${\text{CH}}_{4}+2{\text{H}}_{2}\text{O}⇌{\text{CO}}_{2}+8{\text{e}}^{-}+8{\text{H}}^{+}$$

Equation 1 requires CO_2_, electrons, and protons derived from methane oxidation to leave the cell. For carbon oxidation, the same enzymes of a hydrogenotrophic methanogen are thought to be used in reverse (for reviews, see [100, 102]). All eight electrons derived from carbon oxidation were proposed to enter the membrane-bound methanophenazine pool, and this was proposed to occur at four two-electron steps using three different enzyme modules: HdrDE, Fpo (used twice), and Rnf complexes (Fig. 2A). These complexes have been characterized in methanogens (7, 21, 88, 111) and are present in the ANME-2a genome (107).

Methanophenazine reduction requires two protons, and in the case of the Fpo and Rnf reactions, protons are thought to be derived from the cytoplasm (7, 10, 88). HdrDE is however different as it likely sources protons for methanophenazine reduction from the outside of the cell, the reverse of its “forward reaction” when operating in an energy-conserving redox loop (21, 93). Cumulatively then, although all 8 electrons from methane are thought to be transferred to methanophenazine, only 6 of the protons from reaction 1 would be transferred (by the Fpo and Rnf reactions). 2 protons would remain in the cytoplasm (those from the Hdr reaction), and these protons must be compensated for by chemiosmotic reactions at other steps in the metabolism.

A similar model to that described above can also be imagined for ANME-1, now that they too have been proposed as actors in direct interspecies electron transfer (108) (though the carbon pathway may vary slightly since they appear to lack the gene coding for the methylene-H_4_MPT reductase enzyme: Mer [61]). An Fpo system of the same type thought to be involved in ANME-2 was identified in ANME-1, but an Rnf based mechanism for ferredoxin oxidation was not (61). Going off the idea for “*Ca.* M. nitroreducens” (4) (see below, and Fig. 2B), it could be that ferredoxin is oxidized by electron confurcation involving the oxidation of CoMSH+CoBSH and ferredoxin with reduction of two F_420_. The two H_2_F_420_ produced would later be oxidized by the membrane-bound Fpo complex and result in chemiosmotic ion pumping across the membrane. Alternatively, it could be that the ferredoxin is directly oxidized by the Fpo complex in a similar manner to that proposed for *Methanosaeta thermophila* (110). A similar physiology may also exist for “*Ca.* M. nitroreducens”, since as discussed below, they appear to lack the Rnf complex (see below).

How could the protons and electrons bound by methanophenazine leave the membrane of these methane oxidizing archaea? It is possible to imagine this process occurring in an analogous fashion to what is thought to happen in bacteria in the “porin-cytochrome” model (78)—where the bacterial outer membrane is replaced with the archaeal S-layer, and the porin complex is replaced with the proposed archaeal integral S-layer cytochrome complex. For both electron and proton export out of the membrane, it could be that a homolog of the cytochrome-b HdrE or VhoC proteins may interface with the S-layer bound cytochromes and function as a methanophenazine oxidoreductase. Perhaps there is something like the bacterial CymA, which facilitates proton and electron transfer from quinol to the outside of the membrane (54, 58). Similar to CymA, the proposed protein in methane oxidizing archaea would not be itself proton motive, and the protons derived from methane oxidation would be released from the methanophenazine pool on the outside of the cell (Fig. 2A, top left). Electrons from methanophenazine (MPH_2_/MP: E^0′^=−165 mV [103]) would then be transferred to S-layer cytochromes and finally to extracellular electron acceptors such as partner SRB (57) or other electron acceptors including Fe^III^ oxides (27, 84), where they would presumably be at a sufficiently reducing potential to be able to drive the corresponding reductions, for example the APS/HSO_3_^−^ (E^0′^=−60 mV) and HSO_3_^−^/HS^−^ (E^0′^=−120 mV) couples (99, 102) in SRB.

How many ions are pumped, and how much ATP is synthesized during methane oxidation as depicted in Fig. 2A? There is considerable uncertainty and to be sure, nobody currently knows, however a brief discussion may aid in the discovery of what is yet unknown. The ATP yield of the metabolism will depend on i) the pumping stoichiometry of the complexes indicated (including ATPase), ii) the thermodynamic efficiency of the metabolism (the amount of energy lost as heat), and iii) the intracellular pH and ratio of ATP:ADP in the cell. Considering points i) and ii), the pumping stoichiometries for chemiosmotic coupling complexes exist for homologs when they operate in methanogenic pathways (7, 21, 88) and those numbers can serve as guides, however they are not known for any methane oxidizing archaea. The amount of energy wasted as heat during metabolism, and whether some of these pumping units have variable pumping stoichiometries also remains unknown. One possibility is that these complexes “slip”, which may be a function of the thermodynamic driving potential, as suggested for other molecular motors (5). Regarding point iii), we can consider the energy of ATP formation in a growing cell to be approximately +60 kJ mol^−1^ (85), but may be as low as approximately +40 kJ mol^−1^ for a cell in the stationary phase or in cases in which total coupled reaction energies occur near equilibrium (40, 97, 105). If methane oxidation with sulfate yields ΔG^′m^=−35 kJ mol^−1^ (1 mM reactants) (30), then we can expect about 0.3–0.4 ATP to be synthesized per methane oxidized within a growing ANME cell (recall that half the reaction energy needs to go to the SRB partner). Since approximately 20 kJ mol^−1^ is required in order to pump a single ion across a charged membrane (85), the net chemiosmotic pumping result of Figure 2A is probably the net pumping of a single ion.

### Nitrate-coupled methane oxidation in anaerobic archaea

Although many steps are shared with the above described marine methane-oxidizing archaea relying on syntrophic direct interspecies electron transfer, nitrate-respiring anaerobic methane-oxidizing archaea “*Ca.* M. nitroreducens” (DAMO archaea) have a number of unique features (Fig. 2B). They appear to use menaquinone (MQ: E^0′^=−80 mV [105]) as the membrane-soluble electron carrier, and this, in turn, was proposed to alter overall the bioenergetics, for example the ion-pumping stoichiometry of the Fqo enzyme, where the larger potential difference between MQ and H_2_F_420_ than between MP and H_2_F_420_ could result in the ability to do more work with the reaction (4) (other methane-oxidizing archaea await definitive assignment of the membrane bound electron carrier but are thought to use methanophenazine). In addition, the genomes currently available appear to lack the Rnf complex. In SRB-paired ANME-2, that complex was proposed to be responsible for oxidizing reduced ferredoxin and translocating sodium ions across the membrane (Fig. 2A, top) (57). As an alternative, ferredoxin recycling in “*Ca.* M. nitroreducens” was proposed (4) to use an electron pair-confurcation involving CoMSH+HSCoB and ferredoxin oxidation with two F_420_ reduction (Fig. 2B, top left). This possibility was discussed above in ANME-1 and may also account for ferredoxin recycling in “*Ca.* M. nitroreducens” during iron oxide reduction. The BLZ1 “*Ca.* M. nitroreducens” strain was found to encode for a homolog of an Ech hydrogenase that may also be used for ferredoxin oxidation; however, that protein lacks the two “CxxC” motifs needed to ligate the active site cluster (1, 106) so cannot be considered to be operative in hydrogen metabolism in the organism.

A further difference in “*Ca.* M. nitroreducens” compared to the syntrophic sulfate case is the amount of energy available when using nitrate as terminal electron acceptor. “*Ca.* M. nitroreducens” does not appear to be energetically bound to its partners when in consortium and the partner organism might function for nitrite removal, since methane oxidation coupled to the reduction of four nitrate molecules to nitrite is expected to yield ΔG^′m^=−523 kJ mol^−1^ (4, 17, 30) (1 mM reactants). This compares quite favorably to methane oxidation linked to the SRB value of ~−15–20 kJ mol^−1^ (half the total chemical potential of the reaction: see above). In the case of ANME coupled to SRB, the energetics might be interpreted to fit the observed low growth rate of the organisms. However, even with available energy and an apparent capacity to utilize it, “*Ca.* M. nitroreducens” has also been reported to grow with slow doubling times in the order of weeks (74) and has a similar methane oxidation rate to sulfate-coupled ANME (in the range of fmol CH_4_ [cell*d]^−1^) (35, 67, 68, 74). Perhaps energy yield does not scale directly with doubling times in these organisms or growth rate is limited by some currently unknown factor. Or simply, perhaps growth is governed by the kinetics of Mcr enzyme reaction step which correlates to *in vivo* rates (83).

## Methanogen-methanotroph “switch back”?

Related to the question of methanogen-methanotroph switch back is the process known as “reverse acetogenesis”, which is known to be performed by bacteria in syntrophic relationships with H_2_-consuming partner organisms. In one variety, acetate is consumed using an oxidative acetyl-coenzyme A pathway, and the hydrogen produced is consumed by partner methanogens (36–38, 89, 113, 116). Other examples exist where the syntrophic relationship is with a non-methanogenic H_2_ consumer (19, 115), with acetate being oxidized via an oxidative citric acid cycle (31).

Although some acetate oxidizers have been shown to be truly reversible and function as acetogens (*e.g.* [37, 89]), existing research indicates that methanogens cannot perform net methane oxidation (64, 114). Furthermore, methane-oxidizing archaea appear to be incapable of net methanogenesis (24, 109), and this lack of methanogenesis is explained from a genome perspective since they lack enzymes for H_2_ and methyl compound activation (4, 35, 107). Yet, could a methanogen or a methane oxidizer reverse its central carbon redox reactions by re-arranging or inducing particular sets of proteins that would allow them to conserve energy during the process?

In the case of methane oxidation by methanogens, it might be that they could donate electrons from methane to an inorganic acceptor, since methanogens have previously been shown to reduce elemental sulfur (95), Fe^III^ (11, 14), humics, AQDS (14), and various minerals (46, 47). A recent study reported that *M. acetivorans* formed acetate when methane oxidation was coupled to Fe^III^ reduction; however, methane oxidation with net CO_2_ production was not observed (94). Methane consumption was enhanced with a cell line that expressed the Mcr protein from an ANME-1 genome, and ~140 μmol of methane was oxidized in the course of ~50 μmol of acetate production. A mechanism in which 4 methane are oxidized and 2 CO_2_ are reduced to produce 3 acetate was proposed. Notable points in this metabolism include i) it possibly being facilitated by an electron pair bifurcation reaction in which two CoMSH and CoBSH pairs are oxidized with the concomitant reduction of one ferredoxin and two Fe^3+^ (total movement of four electrons), and ii) the cell model drawn indicated energy conservation by substrate level phosphorylation, not chemiosmosis. This is a very interesting example of apparent methane oxidation for the first time in an isolated methanogenic archaeon and warrants further studies.

The question of reversibility is also applicable to methaneoxidizing archaea: do they have the ability to perform “reverse methanotrophy” and function methanogenically from CO_2_? In the case of ANME-1, ANME-2, and “*Ca.* M. nitroreducens”, they lack the hydrogenase enzymes needed for electron delivery onto CO_2_ (with the exception of the organism described by Haroon *et al.* [35], which appears to have Mvh and Frh which could potentially permit hydrogenotrophic growth by electron pair bifurcation). Yet, perhaps methane oxidizing archaea could reverse their metabolism if grown on a cathodic (electron donating) electrode which could donate sufficiently reducing electrons for the formation of methane. Bacteria are known to grow on electrodes (for reviews see (52, 53)), and methanogens have indeed been shown to be capable of methane formation whilst on cathodes (although growth was not observed) (49); perhaps ANME archaea will be found to be capable of this as well.

## What was first? Forward or reverse?

One interesting area for future research concerns the evolution of ANME archaea. As noted above, different ANME groups are polyphyletic in rRNA and functional gene phylogenies. Since they use some of the same enzyme components as methylotrophic methanogens (*e.g.* Fpo and Rnf), they might be considered to have been derived from methylotrophic methanogens. Perhaps then, the capacity to perform methane oxidation came by way of the ability to shuttle electrons out of the cell. If so, the observation of polyphyletic ANME groups using multiple electron acceptors is an interesting example of convergent evolution to the methane-oxidizing phenotype.

For methane oxidizing archaea proposed to operate through DIET, reductive evolution might be related to the apparent obligate syntrophy of the group, where—once the relationship with an electron consuming SRB was established—the genes associated with methanogenic metabolism (*e.g.* hydrogenases and methyl-compound metabolism enzymes) were lost. This apparent convergence of strategy between the phylogenetically distinct groups of ANME-1 and 2 could result from an energetic gain of efficiency in metabolic coupling via syntrophic DIET in comparison to the passage of a diffusible intermediate (3, 51, 57, 86, 96). On the other hand, for nitrate-reducing “*Ca.* M. nitroreducens”, gene loss may have been facilitated by the acquisition of nitrate and nitrite reductases from bacteria (35). Detailed phylogenetic and biochemical analyses of both the respiratory and C1-metabolizing enzymes in ANME will contribute to ordering the evolutionary events that resulted in the methane-oxidizing phenotype, particularly as our knowledge of organisms with methanogen-like genes and metabolism expands (28, 44, 56). It could be imagined that the ANME phenotype is one of many evolutionary trajectories away from what may have been an ancestral hydrogenotrophic methanogen phenotype (15, 75).

## Perspective

A diversity of enzyme compliments are found within methanogens and acetogens, which accomplish what at first glance looks like the same overall metabolisms respectively— making methane (100, 111), or making acetate (73, 90, 91). Yet the differences in specific enzyme compliments are significant because they influence basic energy metabolism and its yield. These differences may even result in “reverse” energy metabolisms, and it is somewhat surprising that complete energy metabolism reversal can apparently be achieved with only a few changes in the form of electron entry and exit points and the chemiosmotic coupling sites discussed herein (Fig. 1 and 2). Based on the models presented, the main differences between a hydrogenotrophic methanogen with cytochromes and methane-oxidizing archaea are the replacement of the Ech, Vho, and Frh hydrogenases with Fpo, Rnf (or another ferredoxin oxidation mechanism), and a terminal electron release protein in the form of Mhc/Nar (Fig. 1). It may be that one cell type harbors all of these protein complexes and shift its metabolism in a truly reversible manner, similar to some acetogens; however, further *in situ* investigations are needed to test this.

It is likely that in both acetate and methane metabolisms, continued studies will reveal a variety of subtle, yet critical differences in the enzyme modules harbored which allow these microbes to fill various ecological positions while retaining the core carbon pathways (see [44, 56] for recent findings). For both the methane forming and methane oxidizing archaea, a complete understanding of the enzyme compliments which underlie a given physiology is critical to evaluating ecological position and activity. Going further, since the methane-oxidizing archaea are not phylogenetically coherent (monophyletic), the grouping of these organisms by expected physiology only becomes possible with analysis of the enzyme components discussed herein and also by activity-based studies; it cannot be inferred by phylogenetic position alone.

Further enumeration of the enzyme complexes in these cells as well as their expression patterns will allow a more complete understanding of how catalysts and energy converters can be structured and arranged within these cells in order to permit their respective physiologies. A more distant goal will be to relate this information to what may have been the energy conservation modules used in the very first cells, as there are now a number of hypotheses where metabolisms similar to those discussed here are proposed to have been life’s first (29, 69, 82).
